# Gestational Exercise Increases Male Offspring’s Maximal Workload Capacity Early in Life

**DOI:** 10.3390/ijms23073916

**Published:** 2022-04-01

**Authors:** Jorge Beleza, Jelena Stevanović-Silva, Pedro Coxito, Hugo Rocha, Paulo Santos, António Ascensão, Joan Ramon Torrella, José Magalhães

**Affiliations:** 1 Department of Cell Biology, Physiology & Immunology, Faculty of Biology, University of Barcelona, 08028 Barcelona, Spain; jtorrella@ub.edu; 2 Laboratory of Metabolism and Exercise (LaMetEx), Research Centre in Physical Activity, Health and Leisure (CIAFEL), Faculty of Sport, University of Porto, 4200-450 Porto, Portugal; jelena@fade.up.pt (J.S.-S.); pcoxito@fade.up.pt (P.C.); aascensao@fade.up.pt (A.A.); jmaga@fade.up.pt (J.M.); 3 Newborn Screening, Metabolism and Genetics Unit, Human Genetics Department, National Institute of Health Dr Ricardo Jorge, 4000-053 Porto, Portugal; hugo.rocha@insa.min-saude.pt; 4 Faculty of Sport, University of Porto, 4200-450 Porto, Portugal; psantos@fade.up.pt

**Keywords:** maternal exercise, epigenetics, mitochondria

## Abstract

Mothers’ antenatal strategies to improve the intrauterine environment can positively decrease pregnancy-derived intercurrences. By challenging the mother–fetus unit, gestational exercise (GE) favorably modulates deleterious stimuli, such as high-fat, high-sucrose (HFHS) diet-induced adverse consequences for offspring. We aimed to analyze whether GE alters maternal HFHS-consumption effects on male offspring’s maximal workload performance (MWP) and in some skeletal muscle (the soleus—SOL and the tibialis anterior—TA) biomarkers associated with mitochondrial biogenesis and oxidative fitness. Infant male Sprague-Dawley rats were divided into experimental groups according to mothers’ dietary and/or exercise conditions: offspring of sedentary control diet-fed or HFHS-fed mothers (C–S or HFHS–S, respectively) and of exercised HFHS-fed mothers (HFHS–E). Although maternal HFHS did not significantly alter MWP, offspring from GE dams exhibited increased MWP. Lower SOL AMPk levels in HFHS–S were reverted by GE. SOL PGC-1α, OXPHOS C-I and C-IV subunits remained unaltered by maternal diet, although increased in HFHS–E offspring. Additionally, GE prevented maternal diet-related SOL miR-378a overexpression, while upregulated miR-34a expression. Decreased TA C-IV subunit expression in HFHS–S was reverted in HFHS–E, concomitantly with the downregulation of miR-338. In conclusion, GE in HFHS-fed dams increases the offspring’s MWP, which seems to be associated with the intrauterine modulation of SM mitochondrial density and functional markers.

## 1. Introduction

The concept of developmental origins of health and disease reveals the importance of maternal environmental exposure during critical periods of perinatal development, which may affect the risk for offspring of chronic diseases in the short or long term in human and rodent models [1,2,3]. In fact, an adverse in utero environment, in conditions such as maternal malnutrition, might prompt offspring maladaptive responses, leading to persistent physiological and metabolic alterations throughout human life [4]. Accordingly, studies performed in rodents revealed that maternal high-fat diet (HFD) during gestation and lactation impairs glucose regulation and alters insulin sensitivity in adult offspring [5,6,7]. These (patho)physiological consequences in offspring result from progressive malfunctioning of several tissues and organs—including skeletal muscle (SM), a key cornerstone tissue associated with local and systemic important adaptive-related axis [8,9,10]—that are usually perpetuated later in life.

In this context, among many subcellular structures influencing SM adaptive routes, particular emphasis is given to the mitochondrial network, due to its well-known roles in a plethora of processes, including energy production, inter-compartmental redox regulation, ion buffering, as well as apoptotic and auto(mito)phagy signaling intermediates [11]. Moreover, SM mitochondrial turnover and renewal through optimizing or compromising the processes of mitochondrial biogenesis, dynamics, and quality control [8,10] ultimately lead to different (un)fitness phenotypes with consequences in exercise performance, as well as training efficiency even early in life [12,13].

Besides being a master tool to improve athlete’s performance, physical exercise has been considered as an important preventive and therapeutic non-pharmacological strategy against several pathological conditions [14,15], including HFD-induced health-related complications [16], with mitochondrial physiology central to the observed beneficial adaptations [11]. In addition, data has shown that maternal exercise (ME) performed during pregnancy provides health-related benefits not only for mothers, but also for their offspring [17,18,19,20]. In fact, studies on rodents have demonstrated that ME improved offspring’s long-term glucose homeostasis [21,22,23,24] and increased the expression of SM mitochondrial content and biogenesis-signaling markers [25]. Furthermore, ME performed before and during pregnancy may increase the offspring’s volition for self-paced free-running wheel exercise [12]. However, the impact of ME on the offspring’s maximal workload performance (MWP), as well as on mitochondrial-associated metabolic biomarkers is so far unknown. Yet, this concern could potentially be of high interest considering the link between the effectiveness of ME in the offspring intergenerational programming and physical fitness contribution to health outcomes throughout life [26].

Nowadays, gestational exercise (GE), performed by the mother exclusively during the period of pregnancy, is a potential proposed exercise model to improve mothers’ and offspring’s health-related issues [20,27]. Therefore, our intention here was to ascertain whether gestational exercise (GE) mitigates the effects of maternal HFHS-consumption on male offspring’s MWP, and on some SM biomarkers associated with mitochondrial biogenesis and oxidative fitness. These include, among others, the usual biomarkers of mitochondrial density (citrate synthase (CS) activity and mitochondrial DNA (mtDNA) content) [28], key regulatory proteins associated with mitochondrial biogenesis signaling (5′ adenosine monophosphate-activated protein kinase (AMPk), peroxisome proliferator-activated receptor-γ coactivator-1α (PGC-1α), nuclear factor-erythroid factor 2-related factor 2 (NRF2), and mitochondrial transcription factor A (TFAM)), inner mitochondrial membrane proteins known to actively influence electron transport chain activity and oxidative phosphorylation (oxidative phosphorylation subunits (OXPHOS), adenine nucleotide translocator (ANT)), and transmembrane proton gradient and redox environment (uncoupling protein 3 (UCP3)) [11]. Also, microRNAs (miRNAs) were analyzed as important players in the post-transcriptional regulation of gene expression coding for some of these proteins (miR-378a, miR-494, miR-338, mirR-34a) in SM with distinct metabolic phenotypes—the soleus (SOL) and tibialis anterior (TA). We hypothesized that GE attenuates the negative effects of maternal HFHS-consumption on offspring MWP, at least in part, through SM mitochondrial remodeling.

## 2. Results

The present study focused on the effects of GE on MWP and the associated SM markers of oxidative metabolic features in 6-week-old male offspring from dams fed with an HFHS diet. Two different SM were considered to study the impact of maternal environmental exposure on distinct SM phenotypes— the SOL as a muscle with a marked oxidative phenotype, and the TA as a muscle with a heterogeneous constitution and function.

**Figure 1:**
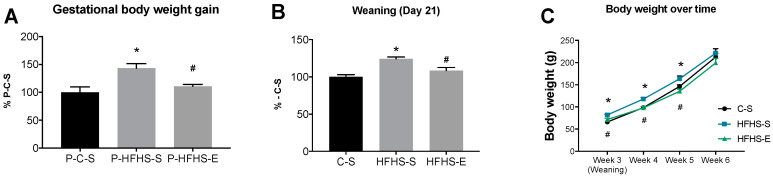
Gestational body weight gain of dams during pregnancy (**A**); offspring body weight at 21 days of age (**B**); and offspring body weight from weaning until 6 weeks of age (**C**). Control sedentary dams (P–C–S); high-fat, high-sucrose sedentary dams (P–HFHS–S); high-fat, high-sucrose exercised dams (P–HFHS–E); offspring from control diet sedentary group (C–S); offspring from high-fat, high-sucrose diet sedentary group (HFHS–S); offspring from high-fat, high-sucrose diet exercised group (HFHS–E), *n* = 6. Data are expressed as the mean ± SEM. Statistically significant differences (*p* ≤ 0.05) are indicated as follows: * vs. C–S, # vs. HFHS–S.

Male offspring of sedentary HFHS-fed mothers had significantly higher body weight (BW) at weaning compared to the offspring of sedentary C-fed mothers, suggesting that GE prevented the adverse effects of maternal HFHS on offspring BW (Figure 1B). Although, the BW differences between groups were maintained throughout the 2 remaining experimental weeks, no significant statistical differences were found in offspring BW in the 6th week (Figure 1C).

As presented in Table 1, the SOL-to-BW ratio was significant lower in the HFHS–S group when compared to C–S (*p* = 0.014; *d* = 1.603), but was restored back to baseline values in offspring from exercised mothers (*p* = 0.030; *d* = 2.070). Still, in the TA, no significant alterations associated with maternal nutrition were found (C–S vs. HFHS–S), while an increase in the TA-to-BW ratio was found in HFHS–E animals compared to their sedentary counterparts (*p* = 0.001; *d* = 2.644). 

Offspring of HFHS–S mothers exhibited higher blood triglycerides compared to their C–S counterparts (HFHS–S vs. C–S; *p* = 0.023; *d* = 2.134); however, GE, despite maternal HFHS feeding, reverted triglycerides levels back to baseline (HFHS–S vs. HFHS–E; *p* = 0.001; *d* = 2.260).

Table 2 shows performance data, namely maximal speed and time to exhaustion. As can be observed, offspring from the HFHS–E group were able to run at higher maximal speed (*p* = 0.009; *d* = 1.110) during a longer period (*p* = 0.012; *d* = 1.052) compared to their HFHS–S counterparts. No differences between groups were found in basal and post-exercise blood lactate and glucose contents.

**Figure 2:**
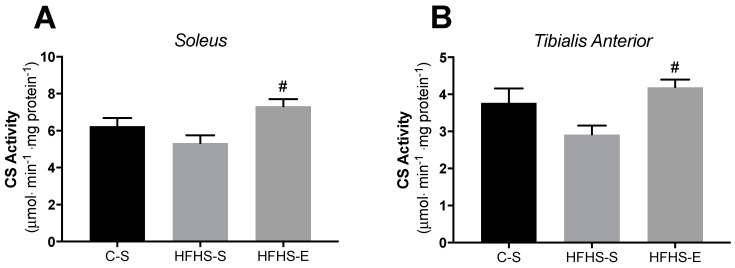
Citrate synthase activity normalized to muscle protein content in the soleus (**A**) and the tibialis anterior (**B**). Offspring from the control diet sedentary group (C–S); offspring from the high-fat, high-sucrose diet sedentary group (HFHS–S); offspring from the high-fat, high-sucrose diet exercised group (HFHS–E), *n* = 6. Data are expressed as the mean ± SEM. Statistically significant differences (*p* ≤ 0.05) are indicated as follows: # vs. HFHS–S. The coefficient of variation in the soleus was: 6.8% for the C–S group; 4.5% for the HFHS–S group; and 4.3% in the HFHS–E group. In the tibialis anterior the coefficient of variation was: 5.7% for the C–S group; 7.1% for the HFHS–S group; and 2.7% in the HFHS–E group.

Figure 2 shows CS activity in the SOL and TA muscles. No significant alterations induced by the maternal HFHS diet were noted in the CS activity of both SM from sedentary dams (HFHS–S vs. C–S). However, offspring from exercised HFHS-fed mothers had higher CS activity in the SOL and TA compared to the offspring of their non-exercised counterparts.

Offspring acylcarnitine fluxes were evaluated in the TA to ascertain the role of maternal diet and exercise on fatty acid catabolism; however, no differences were observed in acylcarnitine levels as a response to maternal diet or exercise.

**Figure 3:**
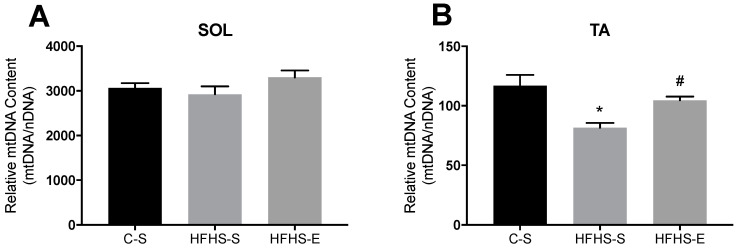
Effects of diet and exercise in mitochondrial DNA content of (**A**) the soleus (SOL) and (**B**) the tibialis anterior (TA) muscles. Offspring from the control diet sedentary group (C–S); offspring from the high-fat, high-sucrose diet sedentary group (HFHS–S); offspring from the high-fat, high-sucrose diet exercised group (HFHS–E), *n* = 6. Data are expressed as the mean ± SEM. Statistically significant differences (*p* ≤ 0.05) are indicated as follows: * vs. C–S, # vs. HFHS–S. The coefficient of variation in the soleus was: 1.9% for the C–S group; 2.4% for the HFHS–S group; and2.4% in the HFHS–E group. In the tibialis anterior the coefficient of variation was: 2.6% for the C–S group; 2.1% for the HFHS–S group; and 1.8% in the HFHS–E group.

As can be deduced from Figure 3, relative mtDNA content was not altered by either maternal exposure to the HFHS diet and/or exercise in the SOL (Figure 3A), while in the TA, the HFHS–S offspring group had a lower mtDNA content than either of the C–S and HFHS–E groups (Figure 3B).

**Figure 4:**
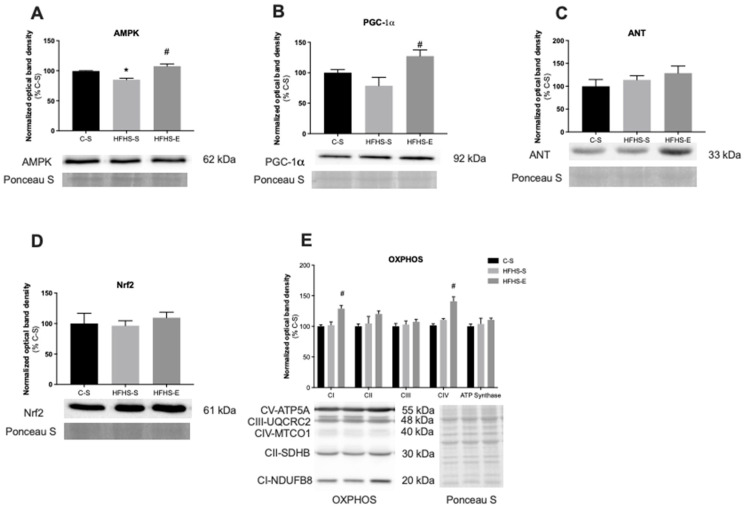
Relative protein content of 5′ adenosine monophosphate-activated protein kinase (AMPk) (**A**); peroxisome proliferator-activated receptor-γ coactivator-1α (PGC-1α) (**B**); and adenine nucleotide translocator (ANT) (**C**). Nuclear factor-erythroid factor 2-related factor 2 (Nrf2) (**D**) and oxidative phosphorylation subunits (OXPHOS) (**E**) in the soleus muscle. Offspring from the control diet sedentary group (C–S); offspring from the high-fat, high-sucrose diet sedentary group (HFHS–S); offspring from the high-fat, high-sucrose diet exercised group (HFHS–E), *n* = 6. Data are expressed as the mean ± SEM. Statistically significant differences (*p* ≤ 0.05) are indicated as follows: * vs. C–S, # vs. HFHS–S.

**Figure 5:**
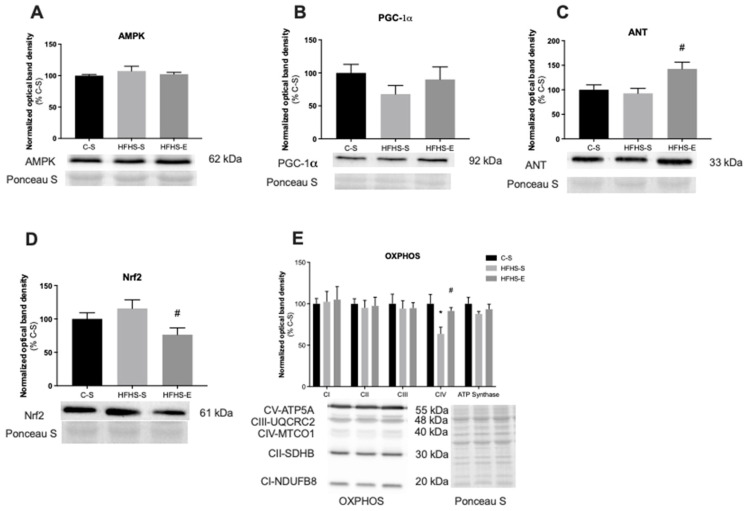
Relative protein content of 5′ adenosine monophosphate-activated protein kinase (AMPk) (**A**); peroxisome proliferator-activated receptor-γ coactivator-1α (PGC-1α) (**B**); and adenine nucleotide translocator (ANT) (**C**). Nuclear factor-erythroid factor 2-related factor 2 (Nrf2) (**D**) and oxidative phosphorylation subunits (OXPHOS) (**E**) in the tibialis anterior muscle. Offspring from the control diet sedentary group (C–S); offspring from the high-fat, high-sucrose diet sedentary group (HFHS–S); offspring from the high-fat, high-sucrose diet exercised group (HFHS–E), *n* = 6. Data are expressed as the mean ± SEM. Statistically significant differences (*p* ≤ 0.05) are indicated as follows: * vs. C–S, # vs. HFHS–S.

Figure 4 and Figure 5 show the relative protein content of some analyzed biomarkers associated with mitochondrial biogenesis and oxidative fitness in SOL and TA, respectively.

As seen in Figure 4A, the SOL AMPk protein content was significantly lower in the HFHS–S offspring group than the C–S group, while an increase in the HFHS–E vs. the HFHS–S group was observed. Despite the AMPk decrease in the HFHS–S group, no alterations were found in the PGC-1α content for the same group. However, the offspring of exercised mothers had a higher SOL PGC-1α protein content (Figure 4B). Interestingly, these alterations in SOL AMPk and PGC-1α contents were not observed when TA was analyzed (Figure 5A,B), while the TFAM protein content was similar in all groups for both muscles.

NRF2 was analyzed as an important regulator of mitochondrial uncoupling and biogenesis in conditions of stress. No alterations were found in the SOL (Figure 4D), while the HFHS–E group showed a decrease in the NRF2 protein content of the TA (Figure 5D). Additionally, the content of OXPHOS complexes I-IV subunits and ATP synthase were also determined. Relative protein content in C-I and C-IV subunits were higher in HFHS–E offspring in the SOL (Figure 4E). In the TA, the C-IV subunit content decreased in the HFHS–S group and was restored back to control values in the HFHS–E group (Figure 5E). No alterations were found in other analyzed subunits within groups from both muscles.

Considering GLUT4 and UCP3 protein contents, neither maternal diet nor GE affected them in any of the analyzed muscles.

Regarding gene expression, although some tendencies are clearly noted, no statistically significant alterations were found in the expression of the selected analyzed genes (Prkaa1; Ppargc1a; Tfam; Cox4i1; Dnm1I; Fis1) both in the SOL and TA.

**Figure 6:**
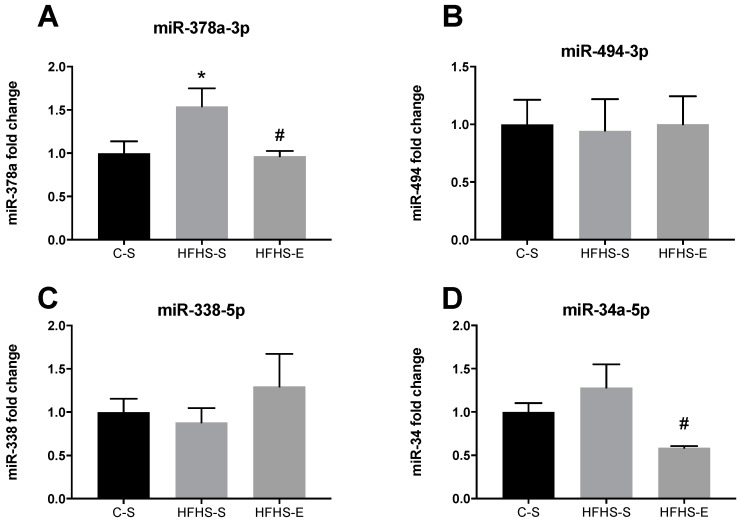
Expression levels of miR-378a-3p (**A**) miR-494-3p (**B**) miR-338-5p (**C**) and miR24a-5P (**D**) in soleus muscle. Offspring from the control diet sedentary group (C–S); offspring from the high-fat, high-sucrose diet sedentary group (HFHS–S); offspring from the high-fat, high-sucrose diet exercised group (HFHS–E), *n* = 6. Data are expressed as the mean ± SEM. Statistically significant differences (*p* ≤ 0.05) are indicated as follows: * vs. C–S, # vs. HFHS–S.

As important players in the post-transcriptional regulation of gene expression coding for some of the proteins, the expression of selected miRNAs potentially involved in mitochondrial biogenesis, fatty acid oxidation, and oxygen consumption were also evaluated. Regarding SOL, the maternal diet significantly elevated the expression of miR-378a. In contrast, offspring from exercised mothers showed a downregulation of miR-378a back to control values, and decreased miR-34a expression (Figure 6A,D).

**Figure 7:**
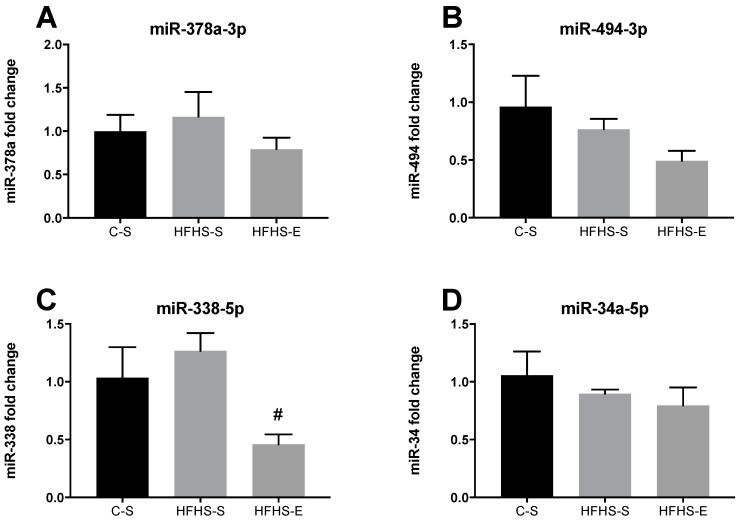
Expression levels of miR-378a-3p (**A**), miR-494-3p (**B**), miR-338-5p (**C**), and miR24a-5P (**D**) in the tibialis anterior. Offspring from the control diet sedentary group (C–S); offspring from the high-fat, high-sucrose diet sedentary group (HFHS–S); offspring from the high-fat, high-sucrose diet exercised group (HFHS–E), *n* = 6. Data are expressed as the mean ± SEM. Statistically significant differences (*p* ≤ 0.05) are indicated as follows: # vs. HFHS–S.

In TA, offspring from the HFHS groups exhibited only a downregulation of miR-338a when compared with the control group (Figure 7C). No other significant alterations were found in the expression of the remaining miRNAs in the TA (Figure 7A,B,D).

## 3. Discussion

### 3.1. Offspring Body Composition

Maternal environmental exposure to unhealthy conditions during critical periods of fetal and perinatal development induces offspring physiological and metabolic changes, including the increased predisposition for the development of chronic diseases [1,2,3,29]. In fact, maternal HFD during gestation and lactation seems to have a negative impact on offspring generation and may increase the propensity to impaired glucose tolerance and insulin sensitivity, as well as the predisposition to obesity development later in the offspring’s life [5,6,30]. In our study, offspring from sedentary HFHS mother had higher BW at weaning, which was in line with previous studies [5,25] and may be a key indicator of increased metabolic disorder risk later in life [31]. Additionally, the impact of maternal lifestyle on the offspring’s BW was maintained until the 6th week of age, in contrast with other studies in which offspring were fed with an HFD after weaning [9,31] suggesting that a postweaning C-diet could eventually prevent obesity later in life [20]. Interestingly, as reported before [25,32], the positive impact of GE during pregnancy on the offspring’s BW was observed not only at weaning, but also until the 6th week of age, as the BW of the HFHS–E group was similar to the control group. In addition, it has been reported that a maternal HFD before and during gestation has deleterious consequences on offspring SM [9,33], such as reductions in SM weight, muscle cross-sectional area, and fiber type composition [34]. In the present study, a maternal HFHS diet decreased the SOL-to-BW ratio, which was restored in the offspring from exercised mothers in parallel with an increase in TA-to-BW ratio when compared to HFHS–S counterparts. Taken together, our data reinforce the role of a maternal active lifestyle, namely of GE, in the modulation of offspring body composition [25].

### 3.2. Skeletal Muscle Mitochondrial Network Physiology

Considering the pivotal role of mitochondrial physiology in SM function, several studies have recently explored the impact of maternal lifestyle on the offspring’s SM mitochondrial network [8,25,27,34,35]. Additionally, it has been reported that maternal HFD induces a decrease in mRNA expression of several biomarkers of mitochondrial biogenesis and dynamics, namely PGC-1α, NRF1, and TFAM, [8,35] associated with downregulation of subunit proteins from C-I and C-II [36]. In the present work, a decrease in TA protein expression in the C-IV subunit was induced by the HFHS diet, although other parameters related to mitochondrial biogenesis and OXPHOS content were not affected by maternal nutrition. The discrepancy between our results and others from previous studies may be related to the diet composition, as our diet was specifically used to induce the condition of gestational diabetes [20,37].

**Figure 8:**
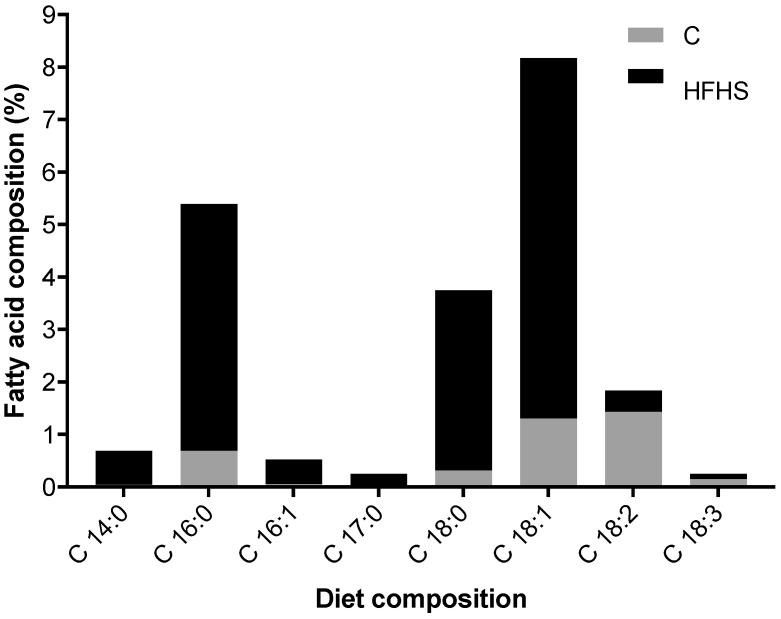
Proportion of fatty acid composition in the maternal diet.

Interestingly, the impact of GE on offspring SM mitochondria was evident. We found an increase in the protein expression of mitochondrial biogenesis biomarkers (AMPk and PGC-1α) in SOL as well as OXPHOS content in SOL (C-I and C-IV subunits) and in TA (C-IV subunits and ANT). Consistently, miR-338, which has been confirmed to inhibit COXIV at the mRNA and protein levels [38], was downregulated in the TA by GE, although surprisingly not in the SOL. Moreover, in accordance with data reported by Simão, et al. [39] and Lee, et al. [40], GE downregulated miR-34a, which is consistent with increased activation of SM AMPk/PGC-1α axis, and mitigates the deleterious impact imposed by a maternal HFHS diet on mitochondrial biogenesis and metabolism-related markers. Being implicated in the regulation of mitochondrial metabolism of SM, miR-378a KO mice developed increased resistance to obesity induced by a high-fat diet and increased efficiency in oxidizing free fatty acids [41]. Accordingly, and in line with the expression of miR-34a, GE induced a downregulation of miR-378a in SOL, but not in TA, which is consistent with the improved oxidative phenotype, although no alterations in SM acylcarnitine levels were found in the offspring of exercised dams when compared to those nonexercised. The overexpression of both miRNAs being associated with mitochondrial dysfunction and concomitant decrease in fatty acid oxidation and oxygen consumption [39,42], our data suggest that GE prevented, at least in part, some mitochondrial impairments through epigenetic regulation. Surprisingly, in contrast with data provided by Yamamoto, et al. [43] suggesting that endurance exercise significantly decreases the content of miR-494 associated with TFAM expression and mitochondrial biogenesis, we did not find a significant decrease in miR-494 and TFAM expression. A clear difference in the experimental sets between these two exercise interventions might eventually explain, at least in part, the differences in the expression of this miRNA. In fact, Yamamoto, et al. [43] evaluated the miRNA expression in the SM of the exercised animals, whilst in our study, data was obtained from the offspring of the exercised dams. Therefore, it seems that maternal exercise is not a sufficiently strong stimulus to influence the expression and role of miR-494 in the intergenerational inherence provided to the offspring’s SM. Differences observed between the SOL and TA could be mainly due to the different SM phenotypic characteristics, as the SOL expresses a prominent oxidative phenotype. Additionally, recent evidence [10] has shown that enhanced mitochondrial biogenesis is associated with increased SM oxidative capacity and with a muscle fiber type shift from glycolytic towards oxidative metabolism in offspring of exercised mothers. Regarding the acylcarnitine profiling, previously we had reported [20] an increase in offspring plasma and liver acylcarnitines (C16, C18, and C18:1) corresponding to those that are predominant in the diet consumed by the mothers (Figure 8). Furthermore, GE was able to restore acylcarnitines back to control levels in plasma and liver. Nevertheless, in this study, although we found an increase in SM long-chain acylcarnitine levels in line with the maternal diet; despite an obvious trend, GE was not able to revert values back to control levels.

### 3.3. Offspring Maximal Workload Capacity

Improvements in endurance capacity, namely in maximal workload performance, induced by different training regimens [28] have been associated with enhancements in mitochondrial function [44,45], and volume density [11,46]. In addition, mice lacking miR-378 through double-KO exhibited better physical performance and improved absolute muscle strength [47]. Although the intergenerational transmission of positive physiological adaptations induced by GE has been poorly described, some evidence suggests that maternal malnutrition may have a negative impact on offspring performance [13] and may be associated with an impairment in mitochondrial network physiology [25,27]. Moreover, Eclarinal et al. [12] reported that offspring from exercised mothers had a higher propensity for physical exercise. Nevertheless, only the work performed by Son et al. [35] has recently shown an improvement in maximal oxygen consumption during exercise in offspring (at weaning) of exercised mothers, as well as a higher exercise time and distance in an acute graded aerobic treadmill test. To the best of our knowledge, the present study is the first to show an improvement in maximal workload test (MWT) in 6-week-old male offspring. Additionally, these improvements in MWT-related maximal speed and time until exhaustion were associated with markers of mitochondrial biogenesis, OXPHOS content, decreases in miRNA-378 expression, as well as with an increase in markers of mitochondrial content and function, such as mtDNA content and CS activity in SM of offspring born from exercised mothers. Some of the alterations found in the present study are in line with mitochondrial remodeling reported by other groups, specifically those related to mitochondrial biogenesis [25] and function [27].

Ultimately, our results suggest that GE may have a vital role in offspring’s SM programming. In fact, SM offspring programming is dependent on phenotypic tissue-specific alterations that seem to be mainly associated with an improvement in the oxidative capacity. Those alterations are potentiated, at least in part, by an epigenetic modulation and consequent intergenerational inheritance.

We assume as limitations of the present study, the absence of an exercise control group and of the report regarding the volume of self-paced free wheel running performed by dams, as this would strengthen comparisons and provide more potential helpful indications to explain the differences between groups.

## 4. Materials and Methods

### 4.1. Animals

All experimental procedures involving animals were performed in accordance with guidelines for Care and Use of Laboratory Animals in Research advised by the Federation of European Laboratory Animal Science Associations (FELASA) and in accordance with the European Convention for the Protection of Animals used for Experimental and other Scientific Purposes (Directive 2010/63/EU). The Ethical Committee of the Institute for Research and Innovation in Health (i3S), University of Porto, and the National Government Authority (Direção Geral de Alimentação e Veterinánia—No 0421/000/000/2018) approved the experimental protocol.

Seven-week-old female Sprague-Dawley rats (150–200 g) were obtained from Charles River Laboratories (L’Arbresle, France) and were randomly divided into two diet groups: control (C) and high-fat, high-sucrose (HFHS) diets. HFHS contained 42% metaolizable energy from fat (vs. 10% in C), 27% from proteins (vs. 20% in C), and 31% from carbohydrates (mainly sucrose, vs. 70% in C with 1% sucrose), with crude fat of 23.1% (vs. 4.1% in C), high cholesterol content, and an increased proportion of long-chain fatty acids (FA) (Figure 8).

**Figure 9:**
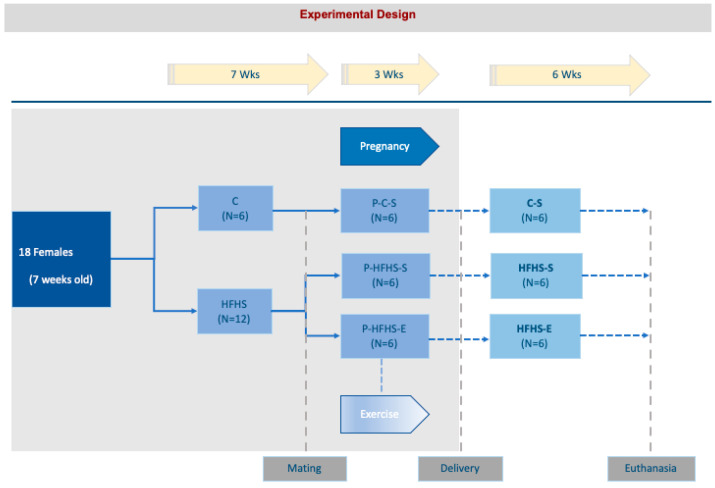
Schematic representation of the experimental design. Seven-week-old female Sprague-Dawley rats were fed with experimental diets—control (C) and a high-fat, high-sucrose (HFHS) diet, and after 7 weeks were mated with sedentary male Sprague-Dawley rats. After 3 weeks of nursing, male offspring were weaned and identified as (*n* = 6 per group): as C–S offspring from P–C–S, HFHS–S offspring from P–HFHS–S, and HFHS–E offspring from P–HFHS–E. Thereafter, offspring were fed with standard laboratory pellet chow and remained sedentary until 6 weeks of age, when they were euthanized.

After 7 weeks on the respective diets (C: E157452–047 or HFHS: D12451(II)mod., Ssniff, Soest, Germany), HFHS dams were divided into two subgroups: HFHS–sedentary dams (P–HFHS–S); and HFHS–exercised dams (P–HFHS–E). The C-diet group remained sedentary throughout the protocol (P–C–S). After the establishment of the three experimental groups (*n* = 6 per group), dams were mated with sedentary C-fed male rats (Charles River, L’Arbresele, France). The morning the vaginal plug was present was considered day 0.5 of pregnancy. After delivery, litter size was reduced to 3 male and 3 female pups to avoid food competition and negative effects on pups’ body mass and development. After 3 weeks of nursing, corresponding male offspring were weaned and identified as (*n* = 6 per group): C–S offspring from P–C–S, HFHS–S offspring from P–HFHS–S, and HFHS–E offspring from P–HFHS–E (Figure 9). Thereafter, offspring were fed with a standard diet and remained sedentary until euthanasia at 6 weeks of age.

Animals were housed in type III-H cages enriched with bedding and nesting material under controlled environmental conditions (21–22 °C; 50–60% humidity), in 12-h light/dark cycles, receiving food (according to respective diet regiments) and water (pH 5.5) ad libitum, in a specific-pathogen-free animal facility at the Institute for Research and Innovation in Health—i3S. During mating and GE protocol during pregnancy, dams were housed in polyethylene type IV cages equipped with a running wheel (perimeter = 1.05 m, Type 304 stainless steel (2154F0106-1284L0106) Tecniplast, Casale Litta, Italy)). All the animal-related procedures during the experimental protocol were blindly performed by independent animal facility technicians.

### 4.2. Physical Exercise Protocol

Exercising dams were submitted to a physical exercise protocol combining a self-paced free-running wheel and endurance treadmill running, as previously established [20]. These animals had 24-h access to a free-running wheel throughout the 3 weeks of the pregnancy period and were exercised on a motor-driven treadmill (LE8700, Panlab Harvard Apparatus, MA, USA) 6 days/week, 20–60 min/day during the dark cycle. The animals were adapted to the treadmill for 5 days before the exercise protocol started. In the first day, rats were placed on the immobile treadmill to adapt to the new device and in the following days, the treadmill was turned on and the speed and duration were gradually increased during the subsequent 4 days.

During the first week of the protocol, GE duration was gradually increased from 20 min/day to 60 min/day (day 1 = 20 min; day 2 = 30 min; day 3 = 40 min; day 4 = 45 min; day 5 = 50 min; day 6 = 60 min) until the speed of 30 cm/s was reached. In the following 2 weeks, the duration was kept at 60 min, but speed was gradually increased until 35 cm/s. Nevertheless, GE intensity was adjusted for each animal in the last few days of pregnancy. Inclination was kept at 0°. Sedentary animals were placed on a non-moving treadmill and housed in cages with blocked wheels to be exposed to the same handling and environmental conditions.

### 4.3. Maximal Workload Test

The maximal workload test (MWT) was performed as previously described [28]. Briefly, offspring were individually evaluated on the treadmill at an inclination of 15°. The test started at a speed of 15 cm/s, after 5 min the speed was set to 30 cm/s, and then increased 3 cm/s every 2 min until maximal workload was achieved, where the animals were no longer able to sustain the treadmill speed. Two criteria were established to confirm maximal workload: the exhaustion of the animal and blood lactate concentration higher than 7 mmol/L. Immediately after the end of MWT and at different time points (1, 3, and 5 min post-MWT), blood samples were collected from the animals using a single blood drop obtained by a puncture in the distal part of the tail until a maximal value of lactate concentration (Lactate Plus Meter, Nova Biomedical, Waltham, MA, USA) was achieved.

### 4.4. Animal Euthanasia and Muscle Harvesting

All animals were fasted overnight for 12 h with access to drinking water before euthanasia. Animals were anaesthetized (induction: 5% isoflurane, 1 L/m O2; maintenance: 2.5% isoflurane, 0.4 L/m O2), right SOL and TA muscles were excised, weighed and immediately frozen in pre-cooled isopentane and stored at −80 °C until further analysis. The blood was collected from inferior vena cava and centrifuged (3000× *g*, 10 min, 4 °C). Obtained plasma was stored at −80 °C until biochemical analysis.

### 4.5. Citrate Synthase Activity

CS activity, as a usual marker of mitochondrial content and oxidative capacity, was measured in SOL and TA homogenates as previously described [28]. Briefly, 10 mg of muscle were homogenized in 100 mL of ice-cold medium (75 mM Tris·HCl, 2 mM MgCl_2_, and 1 mM EDTA (pH 7.6)). The Coenzyme A released from the reaction of acetyl-Coenzyme A with oxaloacetate was measured by its reaction with a colorimetric agent [5,5-dithiobis (2-nitrobenzoate)]. The enzymatic activity was measured spectrophotometrically in two samples at 412 nm.

### 4.6. Muscle Acylcarnitine Levels

For SOL and TA analysis of acylcarnitine levels, an adapted protocol from Petucci et al. [48] was used. Briefly, muscle tissues, homogenized in 80% methanol (1:10 ratio), were centrifuged for 5 min at 14,000 rpm at 10 °C and the supernatant was collected. In a 96-well plate, 140 μL of supernatant and 100 μL of methanol containing deuterated acylcarnitine internal standard solutions (Cambridge Isotope labs, Tewksbury, MA, USA) were added. After drying, using nitrogen flow, the mixture was derivatized to the corresponding butyl esters by incubation with 60 μL of 3 N butanolic HCl at 70 °C for 15 min. The mixture was then dried with nitrogen and reconstituted with 200 μL of 80% methanol for flow injection MS/MS in an API 4000 QTRAP (Sciex, Washington, DC, USA).

### 4.7. Mitochondrial DNA Content

Total DNA was extracted from muscle samples (30 mg of tissue) using GRS Genomic DNA Kit BroadRange (#GK06.0100, GRISP, Porto, Portugal) following the manufacturer’s instructions. Different genes were used to evaluate the relative copy number of mitochondrial DNA (mtDNA) and nuclear DNA (nDNA). For the detection of mtDNA, primers for mitochondrially encoded 16S RNA (Rnr2) were used (forward 5′-AGCTATTAATGGTTCGTTTGT-3′; reverse 5′-AGGAGGCTCCATTTCTCTTGT-3′), whereas for the detection of nDNA, primers for glyceraldehyde 3-phosphate dehydrogenase (GAPDH) were used (forward 5′-GGAAAGACAGGTGTTTTGCA-3′; reverse 5′-AGGTCAGAGTGAGCAGGACA-3′) [49]. Quantitative real-time PCR (RT-qPCR) was conducted using PowerUp SYBR^®^ Green Master Mix (A25742, GRISP, Portugal) in a StepOnePlus^TM^ thermocycler (Applied Biosystems) using paired reverse and forward primers, with mitochondrial and nuclear products being performed separately. All reactions were performed in duplicate and under the same conditions 95 °C for 3 min, 40 cycles of 95 °C for 15 s, and 60 °C for 1 min. The results were presented as mtDNA/nDNA (∆Ct = Ct(nDNA)−Ct(mtDNA); relative mtDNA content = 2 × 2^∆Ct^). 

### 4.8. Protein Semiquantification by Western Blotting

Muscle tissue was homogenized in RIPA lysis buffer (20188, Millipore, Darmstadt, Germany) supplemented with protease and phosphate inhibitor cocktails (P2714, Sigma-Aldrich, St. Louis, MO, USA; 524636, Millipore). After centrifugation (14,000× *g*, 10 min, 4 °C), supernatants were collected and used for semiquantification by Western blotting. Equivalent amounts of muscle tissue were denatured in 2x Laemmli sample buffer and separated by dodecyl sulfate-polyacrylamide gel electrophoresis (SDS/PAGE, 12% gels) [50] and subsequently transferred to PVDF membranes [51]. Membranes were blocked with a blocking agent (non-fat dry milk or BSA in Tris-buffered saline with 0.1% Tween20) to reduce nonspecific binding. After incubation with the following primary antibodies: anti-AMPK (1:1000; ab80039); anti-PGC-1α (1:1000; ab54481); anti-TFAM (1:500; sc-23588); anti-OXPHOS cocktail (1:1000; Abcam, ab110413); anti-ANT (1:1000; sc-70205); anti-Nrf2 (1:500; sc-365949) anti-UCP3 (1:1000; ab10985); and anti-GLUT4 (1:500; sc-53566), and incubation with the following corresponding secondary antibodies: horseradish peroxidase-conjugated anti-goat (1:10,000; sc-2354), anti-mouse (1:10,000; sc-2005), or anti-rabbit (1:10,000; sc-2357), Clarity Western ECL Substrate (Bio-Rad, #1705061) was used for visualization in ChemiDoc-XRS Image System (Bio-Rad), whereas the density of protein bands was determined by using the Image Lab software 6.0.1 (Bio-Rad). For the normalization of eventual differences in protein electrophoresis or transfer to membrane, Ponceau S staining was used [52] and the final data were expressed as the percentage variation of the control values (% C–S).

### 4.9. Real-Time Polymerase Chain Reaction (RT-qPCR)

Total RNA was extracted from muscles by homogenizing 15 mg of tissue using PureLinkTM RNA Mini Kit (12183018A, ThermoFisher Scientific, Waltham, MA, USA) and following the manufacturer’s instruction. Total RNA was then converted to cDNA using Xpert cDNA Synthesis Mastermix (#GK81, GRISP, Porto, Portugal). Real-time PCR was performed using the SYBR Green (PowerUp Sybr Green Master Mix; A25742, ThermoFisher Scientific) method in a StepOnePlus^TM^ (Applied Biosystems). Results were normalized to 18S using fluorescence threshold cycle values (Ct) and expressed and reported as the fold value of the control group C–S group. Specific PCR primer pairs for: *18S* rRNA (forward 5′-CATTCGAACGTCTGCCCTAT-3′; reverse 5′-GTTTCTCAGGCTCCCTCTCC-3′); *Prkaa1* (forward 5′-AGATCGGCCACTACATCCTG-3′; reverse 5′-TCTGGATCTCTCTGCGGATT’); *Ppargc1a* (forward 5′-AAAAGCTTGACTGGCGTCAT-3′; reverse 5′-TCAGGAAGATCTGGGCAAAG-3′), *Tfam* (forward 5′-GCTAAACACCCAGATGCAAAA-3′; reverse 5′-CGAGGTCTTTTTGGTTTTCC-3′); *Cox4i1* (forward 5′-CAGCAGTGGCAGAATGTTGG-3′; reverse 5′-CCACATCAGGCAAGGGGTAG-3′); *Dnm1I* (forward 5′-CCAGGAATGACCAAGGTCCC-3′; reverse 5′-CCTCGTCCATCAGGTCCAAC-3′); *Fis1* (forward 5′-ACTTCTTCTACCCGGAGGCT’; reverse 5′-CTCTACAGGCACTTTGGGGG-3′) were used.

For miRNA analysis, small RNAs were extracted from the rats’ muscles (15 mg) using a mirVana miRNA Isolation Kit (AM1560, Invitrogen, Waltham, Massachusetts, United States) according to the manufacturer’s protocols. The cDNA was obtained using the TaqMan Advanced miRNA Assay (A25576, Applied Biosystems) following the manufacturer’s protocols. The relative expressions of miR-378a-3p, miR-494-3p, miR-34a-5p, and miR-338-5p were determined using the TaqMan Fast Advanced Master Mix (Applied Biosystem) in a StepOnePlus^TM^ (Applied Biosystems). The primers used were assay ID rno481191_mir, rno481191_m; rno481304_mir; rno481068_mir. Relative miRNA expression was reported as fold value of the C–S group.

### 4.10. Statistical Analysis

Sample size estimation was based on previous studies of Pereira et al. [37] and Stevanović et al. [20]. It was calculated that an *n* = 6 per group would suffice to obtain 90% (G*Power, Düsseldorf, Germany) power with *d* = 0.80 effect size, and a confidence interval of 0.95 for the main outcome of the maximal workload test. Data are reported as the mean ± SEM (standard error of the mean). Statistical analyses were performed using GraphPad Prism (version 8.0). One-way analysis of variance (ANOVA) was used for multiple testing and Bonferroni’s correction applied to reduce the probability of a type I error. In all cases, a value of *p ≤* 0.05 was considered statistically significant. Effect size was assessed by calculating Cohen d and interpreted as trivial (<0.2), small (0.2–0.5), medium (0.5–0.8) and large (>0.8) [53].

## Figures and Tables

**Table 1:** Offspring body weight, SM weight, and plasma biochemical analysis.

	C–S	HFHS–S	HFHS–E
Body weight (g)	212.40 ± 8.74	221.20 ± 10.02	199.30 ± 10.23
Soleus/Body Weight (mg/g)	0.47 ± 0.03	0.38 ± 0.02 *	0.45 ± 0.01 ^#^
Tibialis Anterior/Body Weight (mg/g)	1.60 ± 0.03	1.52 ± 0.04	1.74 ± 0.03 ^#^
Glucose (mg/dL)	193.43 ± 11.60	180.20 ± 3.44	211.83 ± 5.78 ^#^
Triglycerides (mg/dL)	64.00 ± 4.78	79.00 ± 0.63 *	51.50 ± 2.14 ^#^

Offspring from control diet sedentary group (C–S); offspring from high-fat, high-sucrose diet sedentary group (HFHS–S); offspring from high-fat, high-sucrose diet exercised group (HFHS–E), *n* = 6. Data are expressed as the mean ± SEM. Statistically significant differences (*p* ≤ 0.05) are indicated as follows: * vs. C–S, # vs. HFHS–S.

**Table 2:** Maximal workload test.

	Max Speed (cm·s^−1^)	T_Exhaustion_ (min)	Lactate_Basal_ (mmol/L)	Lactate_PostEx_ (mmol/L)	Glucose_Basal_ (mg·min/dL)	Glucose_PostEx_ (mg·min/dL)
C–S	48.57 ± 1.52	15.31 ± 2.86	1.81 ± 0.12	7.66 ± 0.24	132.21 ± 3.93	134.00 ± 6.47
HFHS–S	47.50 ± 2.36	15.53 ± 1.33	1.96 ± 0.90	7.50 ± 0.34	135.33 ± 3.00	136.00 ± 6.59
HFHS–E	56.25 ± 2.19 ^#^	20.67 ± 1.50 ^#^	1.94 ± 0.90	7.33 ± 0.80	128.30 ± 3.09	135.40 ± 5.05

Maximal workload test. Final speed achieved at the end of the maximal workload (max speed); time until exhaustion expressed (T_Exhaustion_); basal blood lactate concentration (Lactate_Basal_); highest blood lactate concentration measured at the end of the maximal workload (Lactate_PostEx_); basal blood glucose concentration (Glucose_Basal_); blood glucose concentration measured at the end of the maximal workload (Glucose_PostEx_). Offspring from the control diet sedentary group (C–S); offspring from the high-fat, high-sucrose diet sedentary group (HFHS–S); offspring from the high-fat, high-sucrose diet exercised group (HFHS–E), *n* = 6. Data are expressed as the mean ± SEM. Statistically significant differences (*p* ≤ 0.05) are indicated as follows: # vs. HFHS–S.

## Data Availability

Not applicable.
